# Expression, purification and characterization of the dimeric protruding domain of *Macrobrachium rosenbergii* nodavirus capsid protein expressed in *Escherichia coli*

**DOI:** 10.1371/journal.pone.0211740

**Published:** 2019-02-01

**Authors:** Li Chuin Chong, Hagilaa Ganesan, Chean Yeah Yong, Wen Siang Tan, Kok Lian Ho

**Affiliations:** 1 Department of Biomedical Sciences, Faculty of Medicine and Health Sciences, Universiti Putra Malaysia, UPM Serdang, Selangor, Malaysia; 2 Department of Microbiology, Faculty of Biotechnology and Biomolecular Sciences, Universiti Putra Malaysia, UPM Serdang, Selangor, Malaysia; 3 Institute of Bioscience, Universiti Putra Malaysia, UPM Serdang, Selangor, Malaysia; 4 Department of Pathology, Faculty of Medicine and Health Sciences, Universiti Putra Malaysia, UPM Serdang, Selangor, Malaysia; Instituto Butantan, BRAZIL

## Abstract

*Macrobrachium rosenbergii* nodavirus (*Mr*NV) is the causative agent of white tail disease (WTD) which seriously impedes the production of the giant freshwater prawn and has a major economic impact. *Mr*NV contains two segmented RNA molecules, which encode the RNA dependent RNA polymerase (RdRp) and the capsid protein (*Mr*NV-CP) containing 371 amino acid residues. *Mr*NV-CP comprises of the Shell (S) and the Protruding (P) domains, ranging from amino acid residues 1–252 and 253–371, respectively. The P-domain assembles into dimeric protruding spikes, and it is believed to be involved in host cell attachment and internalization. In this study, the recombinant P-domain of *Mr*NV-CP was successfully cloned and expressed in *Escherichia coli*, purified with an immobilized metal affinity chromatography (IMAC) and size exclusion chromatography (SEC) up to ~90% purity. Characterization of the purified recombinant P-domain with SEC revealed that it formed dimers, and dynamic light scattering (DLS) analysis demonstrated that the hydrodynamic diameter of the dimers was ~6 nm. Circular dichroism (CD) analysis showed that the P-domain contained 67.9% of beta-sheets, but without alpha-helical structures. This is in good agreement with the cryo-electron microscopic analysis of *Mr*NV which demonstrated that the P-domain contains only beta-stranded structures. Our findings of this study provide essential information for the production of the P-domain of *Mr*NV-CP that will aid future studies particularly studies that will shed light on anti-viral drug discovery and provide an understanding of virus-host interactions and the viral pathogenicity.

## Introduction

The giant freshwater prawn or scientifically known as *Macrobrachium rosenbergii* (de Man) is one of the most commercially important farmed aquatic invertebrates in subtropical countries including Malaysia, Indonesia and Thailand [1, 2]. Aquaculture farming has significantly boosted the global aquaculture industry which recorded the highest output value of USD 2.13 billion in 2008. However, the prawn production has declined tremendously since 2012 due to the white tail disease (WTD) that mainly affects prawn hatcheries, and this disease causes great economic loss [1]. The causative agent of WTD is *Macrobrachium rosenbergii* nodavirus (*Mr*NV) which can lead to 100% mortality in larvae, post-larvae, and early juvenile stages of the fresh water prawn [3, 4]. As the name of the disease implies, WTD originates from its milky whitish appearance at the tail of an infected prawn. Other symptoms such as lethargy, anorexia, abdominal muscle opaqueness, as well as a weakened ability to feed and swim [5–7].

*Mr*NV is a non-enveloped virus that belongs to the family *Nodaviridae*. This virus contains two bipartite single-stranded RNA (ssRNA) molecules, known as RNA 1 (3.2 kb) and RNA 2 (1.2 kb), which encode for the RNA dependent RNA polymerase (RdRp) and the viral capsid protein (*Mr*NV-CP), respectively [8, 9]. Currently, there are two established genera in the family of *Nodaviridae*, namely *Alpha-nodavirus* and *Beta-nodavirus*. *Alpha-nodavirus*, one of the causal mortality agents among insect populations, includes black beetle virus (BBV), Boolarra virus (BoV), Flock House virus (FHV), Nodamura virus (NoV), and Pariacoto virus (PaV), while *Beta-nodavirus*, has a major impact on marine fish species, includes barfin flounder nervous necrosis virus (BFNNV), redspotted grouper nervous necrosis virus (RGNNV), striped jack nervous necrosis virus (SJNNV) and tiger puffer nervous necrosis virus (TPNNV) [10]. Interestingly, *Mr*NV, *Penaeus vannamei* nodavirus (*Pv*NV) and covert mortality nodavirus (CMNV) are not clustered in neither *Alpha-* nor *Beta-nodavirus* due to some of their unique characteristics which distinguish them from these two genera [11]. The distinct criterion that differentiates crustacean-infected nodaviruses from the *Alpha-* and *Beta-nodavirus* is the amino acid sequence identity of their capsid proteins. Amino acid sequence alignment revealed that the capsid protein of *Mr*NV shares more than 80% sequence similarity with that of *Pv*NV. However, neither *Mr*NV nor *Pv*NV capsid protein shows similarity higher than 20% with that of *Alpha- or Beta-nodavirus*. Therefore, a new genus known as *Gamma-nodavirus* was proposed [11, 12].

*Mr*NV-CP is a polypeptide comprises of 371 amino acid residues which can be divided into two major domains: the Shell (S) and the Protruding (P) domains, ranging from amino acid residues 1–252 and 253–371, respectively. The full-length and several truncated derivatives of *Mr*NV-CP expressed in *E*. *coli* and insect cells self-assembled into virus like particles (VLPs) [13–15]. The purified recombinant *Mr*NV-CP that assembled into VLPs was used for structural and functional studies. The arginine (Arg) and lysine (Lys)-rich N-terminal region of *Mr*NV-CP is believed to interact with the negatively charged RNA molecules during the viral morphogenesis [16]; the middle portion of *Mr*NV-CP composed of hydrophobic and polar amino acids, whereas the C-terminal region containing the P-domain, has been shown to be involved in host cell attachment and internalization [17]. To understand these mechanisms, a high-resolution atomic structure of the P-domain of *Mr*NV-CP is required. Our previous cryo-electron microscopic analysis revealed that the P-domain is exposed on the surface of VLPs as dimerized protruding blade-like spikes which is distinct from the trimeric spikes found in *Alpha-* and *Beta-nodaviruses* [12]. Recently, an atomic-resolution model of the *Mr*NV-CP was built based on cryo-EM data with an improved resolution at 3.3 Å [18]. However, the local resolution of the dimeric spikes formed by the P-domain of *Mr*NV-CP remained poorer than 4 Å.

In this article, we describe the production of the recombinant P-domain of *Mr*NV-CP in *E*. *coli*. The recombinant protein was then purified to ~90% pure with a two-step chromatography and characterized with size exclusion chromatography (SEC), dynamic light scattering (DLS) and circular dichroism (CD). SEC, DLS and CD analyses demonstrated that the purified *Mr*NV-CP P-domain formed dimer with a hydrodynamic diameter ~6 nm and contained ~68% β-stranded structures. The capability of the P-domain to form a dimer indicates that it mimics the dimeric state of its endogenous form exposed on the viral capsid.

## Materials and methods

### Construction of recombinant plasmid encoding the P-domain of *Mr*NV-CP

The recombinant plasmid, pTrcHis2-TARNA2 [13] encoding the full-length *Mr*NV-CP was extracted using the alkaline lysis method. The nucleotide sequence encoding the P-domain of *Mr*NV-CP (amino acids 253–371) was amplified by PCR using the forward primer (5’ AAT CCT ACA CCA GCC ATG GGC TCA CAG TTA ACT 3’; *Nco*I cutting site is underlined) and the reverse primer (5’ ACG TAA GCT TCG AAT TCG CCC TTA TTA TTG CCG ACG ATA G 3’; *Eco*RI cutting site is underlined). The PCR product was digested with *Nco*I and *Eco*RI (Thermo Scientific, Waltham, USA) and ligated to pTriHis2-TOPO which had been digested with the same restriction enzymes. The ligated plasmid was introduced into *E*. *coli* TOP10 competent cells for protein expression. The nucleotide sequence of the recombinant plasmid was confirmed by DNA sequencing.

### Expression and purification of the recombinant P-domain of *Mr*NV-CP

Expression of the *Mr*NV-CP P-domain in *E*. *coli* was performed according to Goh et al. [13] with some modifications. *E*. *coli* TOP 10 cells harboring the recombinant plasmid was inoculated in Luria-Bertani (LB) broth (500 ml) supplemented with (50 μg/ml) ampicillin and incubated overnight at 37°C with shaking at 200 rpm. The expression of recombinant protein was induced by adding isopropyl β-D-1-thiogalactopyranoside (IPTG, 1 mM) at 30°C for 5 hours. Cells were harvested by centrifugation at 16,200 x*g*, at 4°C. The pelleted cells were resuspended in lysis buffer (25 mM HEPES, 500 mM NaCl, pH7.4) and lysed by adding MgCl_2_ (4 mM), Triton-100 [0.1% (v/v)], freshly prepared lysozyme (0.2 mg/ml), DNase 1 (0.02 mg/ml) and phenylmethylsulfonyl fluoride (PMSF, 2 mM) for 2 hours at room temperature. The cell suspension was then sonicated at 20 kHz for 25 seconds for 10 cycles with 15 seconds intervals between pulses using an ultrasonicator (Qsonica, USA). The crude lysate was recovered by centrifugation at 4°C at 16,200 x*g* for 20 minutes.

The poly-prep chromatography column (Bio-Rad, USA) was packed by adding the profinity IMAC Ni^2+^-charged resin (2 ml) and equilibrated with Tris-HCl buffer (20 mM Tris-HCl, 100 mM NaCl, pH 7.6). The crude lysate was loaded onto the column. Weak binders were removed by Tris-HCl buffer containing imidazole (10 mM) and bound proteins were eluted using Tris-HCl buffer containing Imidazole (100 mM). Eluted fractions were collected for protein analysis using SDS-PAGE [12% (w/v)] and western blot (see below). The positive fractions containing the P-domain of *Mr*NV-CP were pooled, concentrated to ~1.5 ml using a centrifugal protein concentrator with molecular mass cut-off 3 kDa (Merck Millipore, USA) prior to gel filtration chromatography. The concentrated sample was loaded onto a Tris-HCl buffer-equilibrated HiPrep 16/60 Sephacryl S-200 HR column (GE Healthcare, Sweden) mounted on an AKTA Purifier (GE Healthcare, Sweden). Eluents of the column were collected (2 ml per fraction) using a Frac-950 fraction collector at a constant flow rate of 0.5 ml/min. Eluents were analyzed using SDS-PAGE and western blotting. The positive fractions containing the P-domain of *Mr*NV-CP were pooled and concentrated.

### Western blot analysis

The separated proteins on an SDS-polyacrylamide gel were transferred onto a nitrocellulose membrane using a semi dry blotter (Trans-Blot SD, BioRad, USA). The nitrocellulose membrane was then blocked with skimmed milk [10% (w/v)] for 1 hour at room temperature. After three times of washing with TBS-T [50 mM Tris-HCl, pH 7.6; 150 mM NaCl; 0.1% (v/v) Tween 20], the nitrocellulose membrane was incubated with the anti-His monoclonal antibody (Merck, Germany) for 1 hour at room temperature. Afterwards, the nitrocellulose membrane was washed with TBS-T and incubated with the alkaline phosphatase conjugated goat anti-mouse antibody (Merck, Germany; 1:5000 dilutions) for 1 hour at room temperature. The immunoblotted bands were developed by adding 5-bromo-4-chloro-3-indolyl phosphate/nitro blue tetrazolium (BCIP/NBT) in alkaline phosphatase buffer [100 mM Tris-HCl; pH 9.5, 100 mM NaCl, 5 mM MgCl_2_]. Color development was terminated by incubating the nitrocellulose membrane in distilled water and the membrane was allowed to dry at room temperature.

### Estimation of protein molecular mass with size exclusion chromatography

To determine the molecular weight and the multimeric state of the purified P-domain, the gel filtration chromatograms of the P-domain and the protein standard markers [blue dextran (2000 kDa), Ferritin (F; 440 kDa), Aldolase (Ald; 158 kDa), Conalbumin (C; 75 kDa), Ovalbumin (O; 44 kDa), Carbonic Anhydrase (CA; 29 kDa), Ribonuclease A (R; 13.7 kDa), Aprotinin (Apr; 6.5 kDa); GE Healthcare, USA] were overlaid. The molecular mass of the P-domain of *Mr*NV-CP was estimated by comparing the elution profile of the P-domain with the elution profile of the standard markers.

### Quantification of the P-domain of *Mr*NV-CP

The quantity and quality of the P-domain of *Mr*NV at each step of the purification were determined according to Yoon et al. [19]. Briefly, after each step of the protein purification, the P-domain of *Mr*NV-CP was quantified with the Bradford assay [20]. The amount of the P-domain of *Mr*NV-CP from SDS-PAGE gels was measured using the Image J software [21] by calculating protein band intensities. The purity, purification factor and protein recovery were calculated with Eqs 1, 2 and 3, respectively.

$$Purity\;(\%)=\frac{Area\;of\;graph\;of\;the\;P-domain}{Area\;of\;graph\;of\;total\;protein}x\;100\%$$ (1)

$$Purification\;factor=\frac{Purity\;of\;the\;P-domain}{Purity\;of\;the\;P-domain\;in\;crude\;lysate}$$ (2)

$$Recovery\;(\%)=\frac{Amount\;of\;the\;P-domain\;in\;first\;step\;purification\;(SEC)}{Amount\;of\;the\;P-domain\;in\;second\;step\;purification\;(IMAC)}x\;100\%$$ (3)

### Dynamic light scattering (DLS)

The purified protein sample (0.3 mg/ml) was filtered using a 0.2 μm-syringe filter membrane and then loaded into a folded capillary Zeta cell and placed into a Zetasizer Nano ZS (Malvern Instruments) equipped with He-Ne laser (633 nm) to measure the diameter of the P-domain of *Mr*NV-CP. All Zetasizer data were then analyzed using the Zetasizer Software 7.12.

### Estimation of secondary structure

The percentage of the secondary structure of the P-domain of *Mr*NV-CP was estimated using circular dichroism (CD). The buffer of the purified protein sample was exchanged with HEPES buffer (4 mM HEPES, pH 7.6). The protein sample (5 μM) was loaded into a cuvette with 0.1 cm path length and placed in the measurement chamber of a JASCO J-815 CD spectropolarimeter (Jasco International, Japan) after being filtered through a 0.2 μm-syringe filter membrane. The sample was scanned from wavelengths 190 nm to 260 nm, with a speed of 10 nm/min for three times. The data were collected and analysed using the Secondary Structure Estimation Software which was installed in the Spectra Manager version 2. The calculations of the secondary structure elements were carried out based on Yang et al [22] and Reed and Reed [23].

## Results and discussion

### Cloning, expression and purification of the P-domain of *Mr*NV-CP

**Fig 1:**
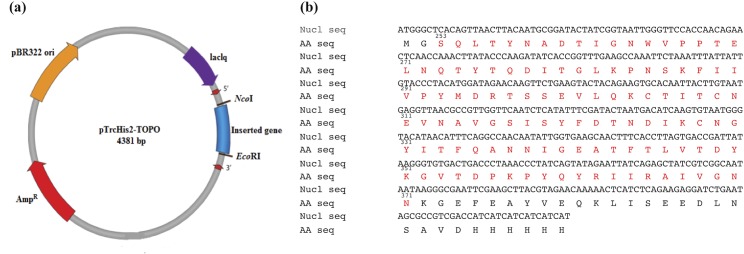
Recombinant expression vector, nucleotide and amino acid sequences of the protruding (P) domain of *Macrobrachium rosenbergii* nodavirus capsid protein (*Mr*NV-CP). (a) Recombinant expression vector containing the nucleotide sequence encoding for the P-domain of *Mr*NV-CP. (b) Nucleotide coding sequence (Nucl seq) for the P-domain of *Mr*NV-CP and the deduced amino acid sequence (AA seq) of the P-domain of *Mr*NV-CP. Numbers above the amino acid sequence refer to amino acid positions of the P-domain *Mr*NV-CP. Amino acid sequence of the P domain is colored in red.

The coding sequence of the P-domain of *Mr*NV-CP was ligated to the expression vector pTrcHis2-TOPO at *Nco*I and *Eco*RI endonuclease restriction sites (Fig 1A). The recombinant plasmid was introduced into *E*. *coli* Top10 cells for protein expression. The recombinant protein comprises 150 amino acid residues including amino acids 253–371 of the P-domain of *Mr*NV-CP, a *myc* epitope and a 6xHis-tag at its C-terminus (Fig 1B). Protein expression was optimized with several parameters including IPTG induction time and induction temperature. A time course study was performed, and an overnight IPTG induction of ~16 hours resulted in the highest protein expression level. However, by considering factors such as the protein stability and time efficiency, induction time of 5 hours was chosen as the most appropriate induction period, which is in good agreement with that reported by Goh et al. [13] for the expression of the full-length *Mr*NV-CP. To obtain an optimum induction temperature, three different induction temperatures (25°C, 30°C and 37°C) were compared. The expression of *Mr*NV-CP P-domain was at its highest expression level at 37°C, followed by 30°C, and the lowest at 25°C. In the expression of full-length *Mr*NV-CP, Goh et al. [13] induced the protein expression at 25°C in order to improve the protein solubility. This temperature mimics the climate of the natural environment where *Mr*NV propagates in the giant freshwater prawn. As a result, we chose 30°C as the most appropriate induction temperature in order to maintain the protein solubility, and at the same time, not compromising the protein expression level.

**Fig 2:**
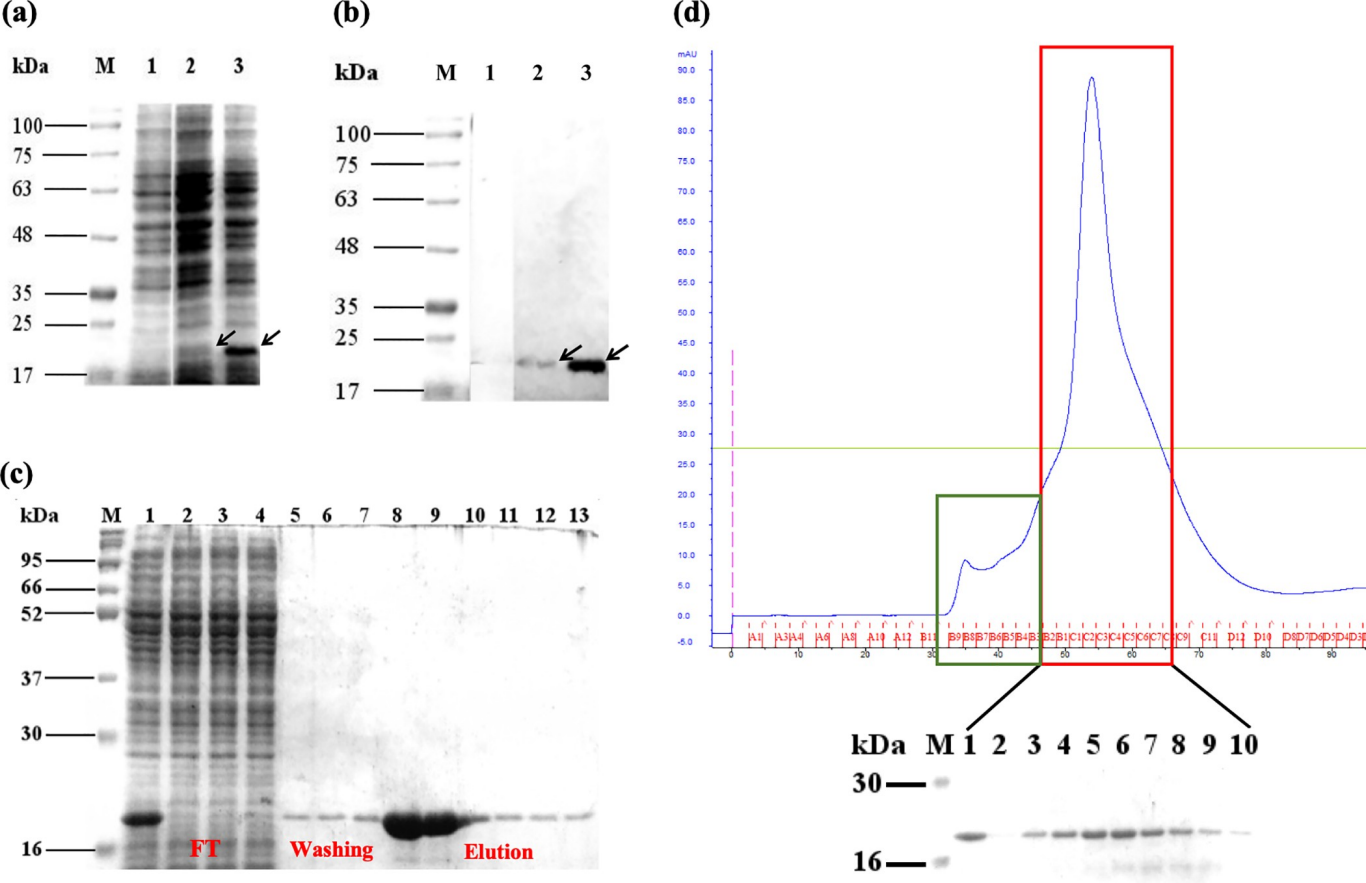
SDS-PAGE and western blot analyses of the recombinant protruding (P) domain of *Macrobrachium rosenbergii* nodavirus capsid protein (*Mr*NV-CP) expressed in *Escherichia coli*. (a) SDS-PAGE, and (b) western blot analysis of the P-domain of *Mr*NV-CP after isopropyl β-D-1-thiogalactopyranoside (IPTG) induction at 30°C. Lane M: protein markers in kDa; lane 1: cell lysate before IPTG induction; lane 2: cell lysate wihout IPTG indution; lane 3: cell lysate after being induced with IPTG for 2 hours. (b) the blot was probed with anti-His monoclonal antibody. Arrowheads indicate additional protein band ~18 kDa in both IPTG induced and uninduced cells. (c) immobilized metal ion affinity chromatography (IMAC) purification. Lane M: protein markers in kDa; lane 1: crude lysate; lanes 2 and 3: flow through; lanes 4–7: fractionated protein samples washed out with washing buffer; lanes 8–13: fractionated protein samples eluted out with elution buffer. (d) Gel filtration chromatogram of the P-domain of *Mr*NV-CP fractionated by HiPrep 16/60 Sephacryl S-200 HR on an FPLC system. The first peak (green box) comprises of elution volume between 33 ml and 45 ml while the second peak (red box) comprises of elution volume between 45 ml and 63 ml. SDS-PAGE analysis of the eluted fractions of the second peak is shown under the chromatogram. Lane M: protein markers in kDa; lane 1: injected sample; lanes 2–10: eluted fractions of the second peak.

The recombinant P-domain of *Mr*NV-CP inclusive of a *myc*-tag and a cleavable 6xHis-tag was expressed as a soluble protein ~18 kDa at 30°C for 2 hours (Fig 2A). Interestingly, the bacterial culture wihout IPTG induction also produced the recombinant P-domain of *Mr*NV-CP as detected in the western blot analysis despite the quantity of this leaky expression was relatively lower than that of the IPTG induced culture. To facilitate protein purification with Ni^2+^ ion chelated nitriloacetic acid (Ni-NTA) immobilized metal ion affinity chromatography (IMAC), a 6xHis-tag was fused to the C-terminal region of the recombinant P-domain of *Mr*NV-CP. As shown in Fig 2C, weakly bound proteins such as host proteins were mostly washed out using a low concentration of imidazole (10 mM) while the 6xHis-tag *Mr*NV-CP P-domain was eluted later with 100 mM imidazole, producing a partially purified protein with protein purity of ~77% (Table 1).

**Table 1:** Purification table of the protruding (P) domain of *Macrobrachium rosenbergii* nodavirus capsid protein (*Mr*NV-CP) expressed in *Escherichia coli*.

	Amount of *Mr*NV-CP P-domain (mg)^a^	Purity of *Mr*NV-CP P-domain (%)^b^	Purification factor^c^	Recovery (%)^d^
Crude lysate	35.6	14.8	-	-
IMAC^e^	12.4	77.0	5.2	34.8
SEC^f^	5.6	88.8	6.0	15.7

^a^ The primary measurement of protein concentration using the Bradford assay by multiplying with the total volume.

^b^ The percentage of the amount of *Mr*NV-CP P-domain against the amount of total protein.

^c^ The ratio of purity of *Mr*NV-CP P-domain in eluted fraction and purity of protein in the crude lysate.

^d^ The amount of *Mr*NV-CP P-domain recovered in relation to the initial amount of protein.

^e^ IMAC; Immobilized metal ion affinity chromatography

^f^ SEC; Size exclusion chromatography

To achieve a better protein purity for downstream analyses such as protein characterizations and structural analysis, another step of protein purification is normally required. In this study, a size exclusion chromatography was performed using HiPrep 16/60 Sephacryl S-200 HR on an FPLC system. The fractionation range of this column is between 5 kDa to 250 kDa. Therefore, this column is suitable to fractionate the P-domain of *Mr*NV-CP with molecular weight ~34 kDa, assuming the *Mr*NV-CP P-domain forms dimers in solution. The size exclusion chromatography fractionated the protein sample containing the P-domain of *Mr*NV-CP into two peaks between 33 ml and 65 ml of elution volume (as indicated by green and red colored boxes in Fig 2D). The first peak (green box) corresponds to the void volume (*V*_*o*_) which did not give a protein band when analyzed with SDS-PAGE whereas the second peak (red box) of the profile gave a protein band with a molecular weight ~18 kDa, which is in good agreement with the molecular mass of the monomer of *Mr*NV-CP P-domain (Fig 2D). Following gel filtration, the purity of the recombinant protein increased to ~89%. Nevertheless, approximately 45% of the protein was lost in the purification process. The purification factor improved from 5.2 in IMAC to 6.0 after the second step of purification. The percentage of recovery, however, reduced from ~35% to ~16% after the gel filtration. During the IMAC purification, host proteins which bound to Ni-NTA column were removed when the protein mixtures were fractionated by a gel filtration column. In addition, certain amount of proteins might be degraded during the process of gel filtration. As a result, it is not surprised to recover only ~16% of the recombinant protein. This is also in accord with Yoon et al. [19] who reported that the recovery of the highly expressed hepatitis B virus capsid was ~20% after the protein was purified with gel filtration chromatography.

### Western blot analysis

**Fig 3:**
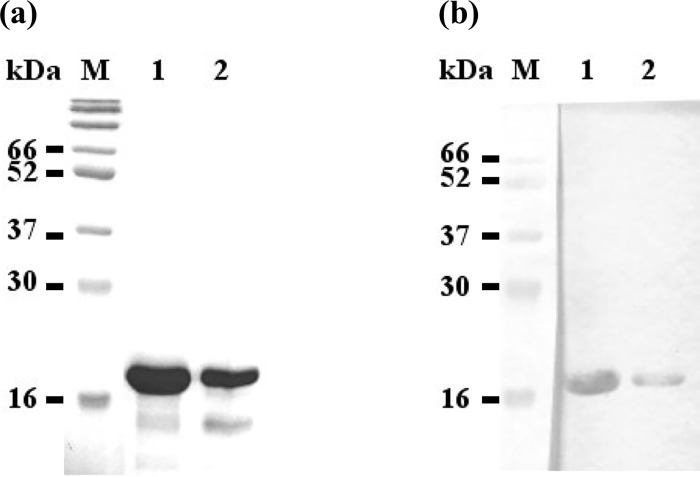
SDS-PAGE and western blot analyses of the purified protruding (P) domain of *Macrobrachium rosenbergii* nodavirus capsid protein (*Mr*NV-CP). (a) SDS-PAGE, and (b) western blot analysis. Lane M: protein marker in kDa; lane 1: concentrated protein sample after IMAC purification; lane 2: concentrated protein sample after gel filtration.

After gel filtration, concentrated protein sample was analyzed by western blotting. Fig 3 shows that the P-domain of *Mr*NV-CP has a molecular mass of ~18 kDa as detected by the anti-His monoclonal antibody. Lower molecular mass protein bands detected by Coomassie brilliant blue (CBB) staining on the polyacrylamide gel (Fig 3A) were not detected by the anti-His antibody, indicating that these smaller protein bands could be the *Mr*NV-CP P-domain fragments without the C-terminus harboring the 6xHis-tag, although the protein was initially purified with an IMAC column. Protein degradation might occur when the protein was concentrated with a centrifugal protein concentrator and other purification processes before the size exclusion chromatography.

### Purified P-domain of *Mr*NV-CP exists homogenously as a dimeric form

**Fig 4:**
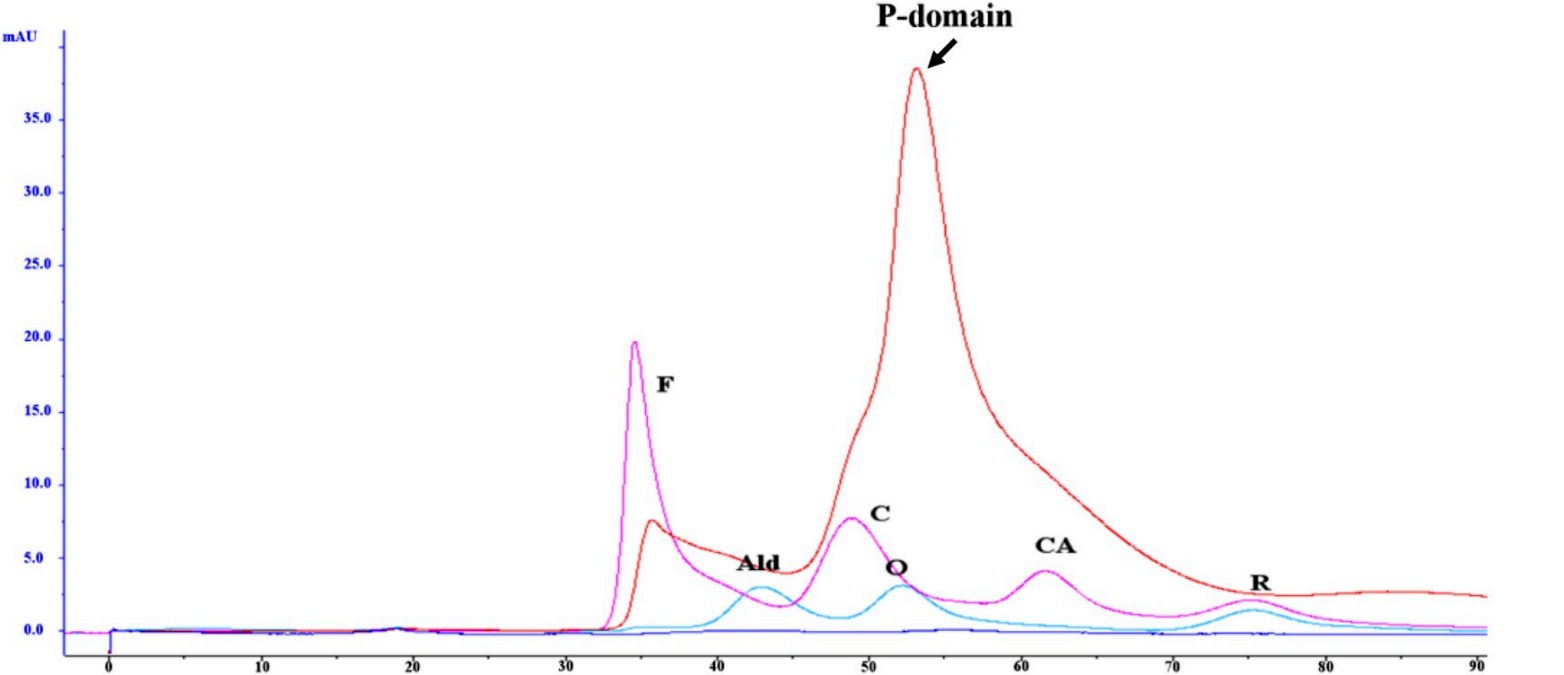
Determination of the multimeric state of the protruding (P) domain of *Macrobrachium rosenbergii* nodavirus capsid protein (*Mr*NV-CP) using gel filtration. The protein fractionation profile of native protein markers and the P-domain of *Mr*NV-CP fractionated by HiPrep 16/60 Sephacryl S-200 HR. Peaks of each protein marker are indicated [Abbreviation: F (Ferritin; M_r_ 440 kDa), Ald (Aldolase; M_r_ 158 kDa), C (Conalbumin; M_r_ 75 kDa), O (Ovalbumin; M_r_ 44 kDa), CA (Carbonic Anhydrase; M_r_ 29 kDa), R (Ribonuclease A; M_r_ 13.7 kDa)]. Red line indicates the chromatogram of the P-domain of *Mr*NV-CP. Purple and blue lines indicate protein markers’ chromatograms. M_r_ indicates relative molecular mass.

**Fig 5:**
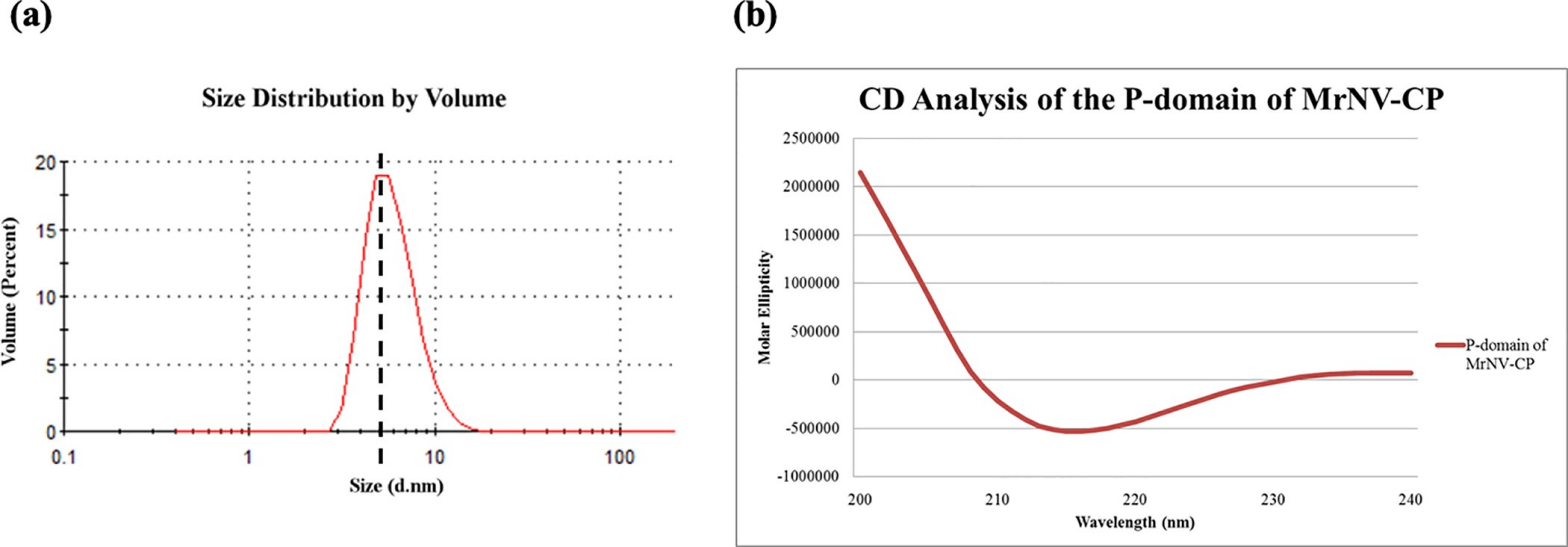
Biophysical characterizations of the protruding (P) domain of *Macrobrachium rosenbergii* nodavirus capsid protein (*Mr*NV-CP). (a) Dynamic light scattering (DLS) analysis of the P-domain of *Mr*NV-CP. The dotted line indicates the diameter of the P-domain. (b) Circular dichroism (CD) spectra of the P-domain of MrNV-CP scanned from near-UV wavelengths 240 nm to 200 nm. The CD spectra display the unique characteristic of beta-stranded structures, indicated by negative bands at wavelength 218 nm.

The molecular mass and multimeric state of the P-domain of *Mr*NV-CP were estimated by overlaying the gel filtration chromatograms of the P-domain and the standard markers. The overlaid chromatograms (Fig 4) shows that the peak containing the P-domain is located between the peaks for carbonic anhydrase (CA, 29 kDa) and ovalbumin (O, 44 kDa), indicating that the P-domain may exist in the form of dimer with a molecular mass of ~36 kDa. This is in good agreement with the cryo-EM analysis reported by Ho et al. [12] who reported that the P-domain of *Mr*NV-CP indeed self-assembled to form dimers. The purified P-domain was then characterized by dynamic light scattering (DLS). As shown in Fig 5A, the purified P-domain of *Mr*NV-CP has a hydrodynamic diameter ~6 nm. The measured diameter of the dimeric particle corresponded well with the cryo-EM structure showing that the blade-shaped spike formed by the P-domain of *Mr*NV-CP is around 4.7 nm in both width and height. It is believed that in the absence of the S-domain, the dimeric conformation of the P-domain alone is possibly distinct from that observed in cryo-EM analysis. In addition, the diameter of a particle determined by DLS is normally bigger than that measured by EM due to the fact that ions in solution associate with the particle and contribute to the size determined by DLS [24].

### P-domain of *Mr*NV-CP is mainly composed of beta-stranded structures

**Fig 6:**
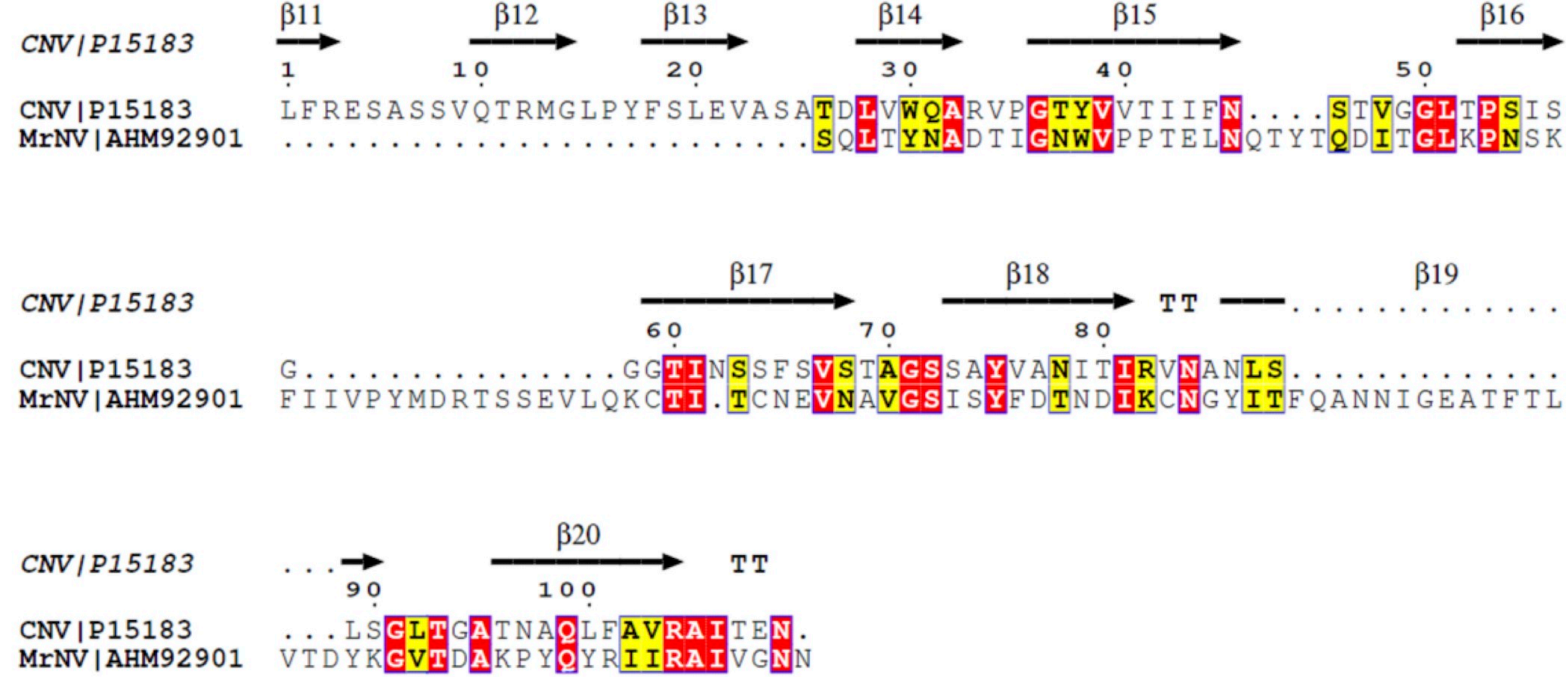
Pairwise sequence alignment of the protruding (P) domain of *Macrobrachium rosenbergii* nodavirus capsid protein (*Mr*NV-CP) and cucumber necrosis virus (CNV). The homology model used was cucumber necrosis virus (CNV). Beta-strands, turns and gaps are indicated by arrows, TT letters and dotted lines, respectively. Identical and similar amino acid sequences are highlighted in red and yellow, respectively.

Circular dichroism (CD) analysis was carried out to estimate the content of the secondary structures of the recombinant P-domain. Fig 5B shows the CD spectra scanned from near-UV wavelength 240 nm to 200 nm. The CD spectra showed that the protein contains a high percentage of beta-stranded structures, which are indicated by the negative bands around 218 nm–a characteristic spectrum for beta-stranded structures [25]. Other unique features for alpha-helical and floppy structures are not observed in the spectra. Based on Reed and Reed [23], the P-domain contained ~67.9% beta-stranded structures and 32.1% turns and unstructured loops. *In silico* analysis performed using Phyre 2 identified the crystal structure of cucumber necrosis virus (CNV) (PDB code: 4LLF [26]) as the closest homologous template to *Mr*NV-CP, the same structure was used as a template to model a 3D structure of *Mr*NV capsid [17, 18]. Amino acid sequence comparison between the P-domain of *Mr*NV and CNV (amino acids 267–380) showed 19% identity and 27.5% similarity, respectively (Fig 6). The P-domain of the crystal structure of CNV contains 57% of beta-stranded structure and also has no alpha-helical structure. Structural similarity for protein pairs with amino acid sequence identity between 20–35% is often referred to as in the ‘twilight zone’, as the likelihood that protein pairs with sequence identity below 25% to have similar structures is less than 10% [27, 28]. In the present study, structural modeling using CNV as a template successfully generated a model with a confidence score of 98% (S1 Fig). The model covers 90% of the amino acid sequence of the P-domain of *Mr*NV-CP. The result suggests that the P-domain of CNV could represent a homologous template for future structural studies of the P-domain of *Mr*NV-CP.

## Conclusion

The P-domain of *Mr*NV-CP was expressed as a soluble protein in *E*. *coli*. The recombinant P-domain formed dimers with a hydrodynamic diameter ~6 nm which resemble the full-length capsid protein. Our current data provide crucial biophysical parameters for downstream analyses of the P-domain of *Mr*NV-CP such as structural analysis using X-ray crystallography or nuclear magnetic resonance. Availability of a high-resolution 3D structure of the P-domain is essential to elucidate the mechanisms involved in host cell recognition and penetration.

## Supporting information

S1 Fig Putative structure of the protruding (P) domain of *Macrobrachium rosenbergii* nodavirus capsid protein (*Mr*NV-CP). The model was generated by program Phyre 2 using the P-domain of cucumber necrosis virus (CNV; PDB code 4LLF) as a template. The confidence score of this model is 98% over 90% of the amino acid sequence of the P-domain of *Mr*NV-CP.
(TIFF)
