# Supplementary figures and images for: Expression, purification and characterization of the dimeric protruding domain of Macrobrachium rosenbergii nodavirus capsid protein expressed in Escherichia coli
Source: PLoS One. 2019 Feb 1;14(2):e0211740. doi: 10.1371/journal.pone.0211740 (PMC6358098; doi:10.1371/journal.pone.0211740)

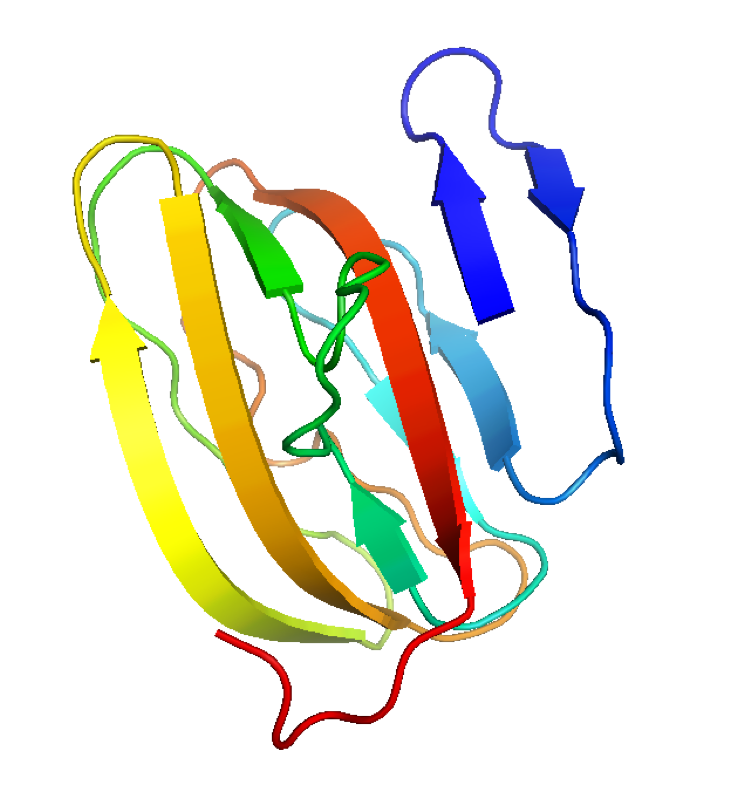

Supplement: S1 Fig — The model was generated by program Phyre 2 using the P-domain of cucumber necrosis virus (CNV; PDB code 4LLF) as a template. The confidence score of this model is 98% over 90% of the amino acid sequence of the P-domain of MrNV-CP. (TIFF) [file pone.0211740.s001.tiff]
